# Serotonin Signaling and Vascular Reactivity in Cardiometabolic Disease and Cardiac Surgery

**DOI:** 10.3390/cells15141265

**Published:** 2026-07-14

**Authors:** Alexander Joseph, Jared Hsu, Bryan Han, Shawn Kant, Lucian Lozonschi, Frank Sellke, Jun Feng

**Affiliations:** 1 Rhode Island Hospital, Brown University Health, Warren Alpert Medical School, Brown University, Providence, RI 02903, USA; alexander_joseph@brown.edu (A.J.); shawn_kant@brown.edu (S.K.); frank_sellke@brown.edu (F.S.); 2 Morsani College of Medicine, University of South Florida, Tampa, FL 33612, USA; jared391@usf.edu (J.H.); bryanhan@usf.edu (B.H.); lozonschi@usf.edu (L.L.)

**Keywords:** serotonin, 5-hydroxytryptamine, 5-HT, vasomotor tone, microcirculation, cardioplegic arrest, cardiopulmonary bypass, cardiovascular surgery, diabetes, metabolic syndrome, myocardial ischemia

## Abstract

**Highlights:**

**What are the main findings?**

- Serotonin-mediated vasomotor dysfunction is mechanistically distinct in metabolic syndrome, diabetes, hypertension, myocardial ischemia, and post-CPB states, driven primarily by altered 5-HT1B and 5-HT2A receptor expression with concurrent COX-2 upregulation.
- Preexisting diabetes and uncontrolled hypertension amplify post-CPB serotonergic vasospasm in tissue-bed-specific patterns mediated by 5-HT1B signaling.

**What are the implications of the main findings?**

- Receptor-selective serotonin antagonism, particularly of 5-HT1B, is a viable therapeutic target for perioperative vasomotor protection in high-risk cardiac surgery patients.
- Routine perioperative SSRI use does not appear to increase CPB-related bleeding or mortality, refining preoperative medication management for an increasingly prevalent comorbidity.

**Abstract:**

Serotonin (5-hydroxytryptamine, 5-HT) is a vasoactive amine and important regulator of vascular tone. Aberrant serotonergic activity contributes to vascular pathology in multiple cardiovascular disease states, including hypertension, diabetes, metabolic syndrome and chronic myocardial ischemia. Serotonin also plays a vital role in postoperative vascular dysfunction after cardioplegic arrest with cardiopulmonary bypass (CP/CPB). Perioperative microvascular dysfunction following CP/CPB manifests as an increased propensity for vasospasm or vasoplegia, with different effects in different tissue beds (coronary, skeletal muscle, pulmonary and systemic microcirculations). Given the importance of serotonin as a modulator of vasogenic responses, we present a selected narrative survey of the current literature concerning the effects of altered serotonergic activity on vascular dysfunction in the context of CP/CPB and other cardiometabolic diseases. This review begins with a discussion of normal serotonin biology and signaling pathways, then moves into disease-specific discussions covering five major areas of focus: metabolic syndrome, diabetes, hypertension, myocardial ischemia and CP/CPB.

## 1. Introduction

First described in 1948, serotonin (5-hydroxytryptamine, 5-HT) is a tryptophan-derived neurotransmitter with major central and peripheral nervous system activity [1]. The majority of serotonin present in the human body is actually found outside of the nervous system, where it has a wide range of functions in vascular, platelet, and gastrointestinal physiology [2].

Serotonin has a variety of known vasoactive effects derived from its interactions with endothelial cells, vascular smooth muscle, and platelet-derived factors. However, despite this, there is a limited overall understanding of the role serotonin plays in several cardiovascular disease states, such as diabetes, hypertension, metabolic syndrome, and ischemia–reperfusion-related injury. This is even more so in the context of cardiac surgery involving cardiopulmonary bypass.

Hence this is the purpose of this focused narrative review, which will first define the current landscape of serotonin biology as it relates to cardiovascular function and common disease states through examining a combination of human and animal studies. We will then extend these findings forward to highlight gaps in the existing literature, and avenues for further investigation. We will also suggest potential therapeutic targets involving serotonergic signaling for patients with cardiometabolic disease, and for protecting vascular function perioperatively in patients undergoing cardiac surgery involving cardiopulmonary bypass.

## 2. Serotonin Receptors and Receptor Physiology

Fifteen known serotonin receptors have been cloned and isolated in the human body [3]. The majority signal through G-protein-coupled receptors (GPCRs) that modulate cyclic AMP or inositol triphosphate depending on subtype [4].

The 15 distinct 5-HT receptor subtypes [5] mediate diverse physiological functions including appetite, behavior, metabolism, gastrointestinal secretion/motility, and vascular tone [6,7,8]. Seven of the 5-HT receptor subtypes (5-HT1B, 5-HT1D, 5-HT2A, 5-HT2B, 5-HT4, and 5-HT7) have been reported to be vasoactive across animal species and vessel types [9,10]. In humans, 5-HT1 and 5-HT2 subtypes mediate vascular contraction in isolated coronary arteries [11,12,13], cerebral arteries [14] and femoral veins [15]. Likewise, 5-HT1 and 2 receptors have additional roles in microvascular inflammation, and heart rate control [16,17]. The 5-HT2A subtype is the predominant serotonergic mediator of vascular contraction in gastrointestinal, umbilical and peripheral vessels in cattle and sheep [9,18,19]. Evidence for 5-HT4 in the vasculature is more sparse, though 5-HT4 upregulation has been implicated in arrhythmia and altered contractility [20]. The 5-HT7 receptor is implicated predominantly in venodilation [21]. Table 1 summarizes the cardiovascular actions of these receptor subtypes.

## 3. Serotonin Biology and Metabolic Syndrome

### 3.1. Peripheral Serotonin Biology and the Tph1/Tph2 Dichotomy

Although serotonin was first characterized as a central neurotransmitter, more than 90% of total body serotonin is synthesized peripherally by enterochromaffin (EC) cells of the gut [33]. Two tryptophan hydroxylase isoforms govern serotonin synthesis: Tph1, expressed predominantly in non-neuronal cells, generates the circulating peripheral pool; Tph2, in contrast, has been classically restricted to the raphe nuclei and enteric nervous system, and generates the central pool [34]. Because serotonin does not cross the blood–brain barrier, the two pools are biochemically independent under physiologic conditions. Recent work has complicated this dichotomy. Park and colleagues demonstrated that obesity-induced hyperinsulinemia in mice upregulates Tph2 expression in adipocytes, contributing to local adipose serotonin production and systemic metabolic dysfunction [35]. This finding suggests that Tph1/Tph2 compartmentalization is context-dependent and that obesity drives a previously unappreciated peripheral Tph2 axis. Peripheral serotonin contributes to glucose homeostasis, lipid metabolism and energy expenditure through tissue-specific 5-HT receptor-expression patterns [36,37]. The relevant cardiometabolic signaling cassettes are summarized below.

### 3.2. Adipocyte, Hepatic and Pancreatic β-Cell Serotonin Signaling

Adipose tissue is both a source and a target of peripheral serotonin. Adipocyte-derived 5-HT, acting through 5-HT2A and 5-HT2B receptors, suppresses brown adipose tissue thermogenesis and uncoupling protein-1 (UCP-1) expression, contributing to weight gain in obese mice; adipocyte-specific Tph1 deletion protects against diet-induced obesity by augmenting brown fat thermogenesis [38]. In visceral white adipose tissue, 5-HT2B signaling on macrophages drives inflammation and insulin resistance; Choi and colleagues showed that pharmacologic or genetic disruption of HTR2B in murine visceral adipose tissue improves obesity-related insulin resistance [39].

In hepatocytes and hepatic stellate cells, gut-derived serotonin signals through 5-HT2A and 5-HT2B to promote hepatic steatosis, gluconeogenesis and fibrogenesis. Choi and colleagues demonstrated, in mice models, a gut–liver serotonin axis in which intestinal Tph1-derived 5-HT drives hepatic steatosis via hepatocyte HTR2A; gut-specific Tph1 knockout protected against high-fat-diet-induced fatty liver [40]. Comprehensive reviews by Yoon et al. and Redensek et al. synthesize the role of central and peripheral 5-HT in metabolic dysfunction-associated steatotic liver disease (MASLD) in both in vitro human and mouse models, implicating 5-HT2B as a particularly attractive antifibrotic target, with peripherally restricted 5-HT2B antagonists now advancing through medicinal chemistry development for MASH [41,42].

In pancreatic β-cells, serotonin is co-stored with insulin in secretory granules and is released alongside insulin during glucose stimulation. Intracellular serotonin participates in β-cell function not only through receptor signaling but also through a post-translational modification called serotonylation, in which transglutaminase-2 covalently attaches serotonin to glutamine residues on small GTPases including Rab3A and Rab27A, modulating insulin granule trafficking [43]. Yoo and Joo demonstrated that nanomolar 5-HT in INS-1E cells, derived from rat insulinomas, modulates insulin synthesis and secretion through TGase2-mediated Rab3A serotonylation, ERK/Akt phosphorylation, and changes in Bcl-2 and superoxide dismutase expression [43]. β-cell expansion during pregnancy is also driven by serotonin acting through 5-HT2B in an autocrine fashion [44].

Skeletal muscle has more recently emerged as an additional peripheral 5-HT target. Park and colleagues reported that muscle-specific Tph1 or Htr2b deletion in mice on a high-fat diet improved glucose tolerance, increased AKT and AMPK phosphorylation, and improved insulin sensitivity; pharmacologic 5-HT2B inhibition reversed palmitate-induced insulin resistance in cultured myotubes [45]. This work extends the peripheral 5-HT–HTR2B axis from adipose tissue and liver to skeletal muscle and identifies HTR2B as a potential pharmacologic target for insulin resistance.

### 3.3. Druggable Peripheral Serotonin Targets in Metabolic Disease

Two pharmacologic strategies have emerged. The first is inhibition of peripheral serotonin synthesis with peripherally restricted Tph1 inhibitors, of which telotristat ethyl (LX1606) is the prototype. Telotristat is FDA-approved for carcinoid syndrome diarrhea and reduces circulating 5-HT by inhibiting EC-cell Tph1 [46]. Although no phase II/III trial has yet tested telotristat in metabolic disease, preclinical work demonstrates that Tph1 inhibition phenocopies adipocyte Tph1 knockout in protecting mice from diet-induced obesity and hepatic steatosis [38]. Awasthi and colleagues recently reported that telotristat enhances chemotherapeutic efficacy in cholangiocarcinoma models, broadening the translational interest in peripheral 5-HT depletion beyond metabolic disease [47].

The second strategy is selective receptor antagonism. Sarpogrelate, a 5-HT2A antagonist approved in Japan and South Korea (but not in the United States) for peripheral arterial disease, has demonstrated favorable effects on vascular function, cardiomyocyte subcellular biology and clinical outcomes in diabetic patients (discussed in Section 3) [29,48,49]. Peripherally restricted 5-HT2B antagonists are in active medicinal chemistry development for liver fibrosis and pulmonary arterial hypertension [42,50]. A 2025 systematic review of 22 human studies on serotonin in obesity confirms the translational interest in peripheral 5-HT modulation, though no agent has yet achieved approval for a metabolic indication [51].

### 3.4. GLP-1 Receptor Agonists and Serotonin Crosstalk

Given the now-dominant role of GLP-1 receptor agonists (GLP-1 RAs) in cardiometabolic practice, the intersection between GLP-1 and serotonergic signaling merits discussion. Three mechanistic threads are relevant. First, EC-cell-derived serotonin and L-cell-derived GLP-1 are produced in adjacent intestinal epithelial cells, and Vanslette and colleagues demonstrated bidirectional crosstalk in mouse models in which 5-HT4 agonism promotes L-cell differentiation and GLP-1 secretion, while liraglutide stimulates intestinal 5-HT release [52]. Second, in the central nervous system, GLP-1 RAs decrease hypothalamic 5-HT2A receptor expression, and pharmacologic 5-HT2A antagonism attenuates GLP-1 RA-induced weight loss in mice [53]. Third, GLP-1 RAs improve endothelial function and arterial stiffness in patients with type 1 and type 2 diabetes [54,55]. Whether any of these effects depend on serotonergic signaling remains untested in humans, but a network meta-analysis of 38 randomized controlled trials in 2065 type 2 diabetes patients confirmed that GLP-1 RAs and SGLT-2 inhibitors significantly improve flow-mediated dilation and pulse wave velocity versus placebo and recent real-world cardiovascular outcome data demonstrate comparable cardioprotection from semaglutide and tirzepatide in more than 800,000 type 2 diabetes patients [54,56]. The mechanistic dissection of serotonergic versus non-serotonergic contributors to GLP-1 RA cardiovascular benefit remains an open research priority.

### 3.5. SSRIs and Metabolic Disease Markers

Selective serotonin reuptake inhibitors (SSRIs) may modestly improve cardiometabolic markers in patients prescribed them for psychiatric indications, with small reductions in total cholesterol and triglyceride levels and blunted platelet aggregation. Corresponding to this observation, certain 5-HT2A polymorphisms are associated with cardiometabolic disease markers: the 5-HT2A, −438 GG and 2416 TT genotypes have been associated with an increased incidence of metabolic syndrome and hypertension [57]. These epidemiologic observations are confounded by depression itself as a cardiometabolic risk modifier; mechanistic studies will need to distinguish receptor genotype effects from background serotonin tone effects.

## 4. Serotonin and Vascular Tone in Metabolic Syndrome and Diabetes

### 4.1. Survey of Animal Models

Large-conduit coronary artery responses to 5-HT differ from those of smaller coronary arterioles [58]. In pigs, coronary arteries and arterioles larger than 90 microns constrict in response to serotonin, while arterioles smaller than 90 microns dilate [59]. Animal models of metabolic syndrome and obesity demonstrate markedly increased serotonin-mediated vascular contractile responses or decreased arteriolar endothelium-dependent relaxation [60]. Serotonin-induced contractions of coronary arterioles harvested from pigs fed a diet designed to induce metabolic syndrome were more pronounced than those harvested from control (lean) pigs [61]. These contractions were significantly inhibited in the presence of phospholipase A2, cyclooxygenase and thromboxane synthase inhibitors, consistent with the role of these downstream effectors in serotonin contraction [61]. In arterioles from normal pigs, serotonin produced dose-dependent vasodilation that was abolished by endothelial denudation. In arterioles from atherosclerotic pigs, serotonin produced dilation only at the highest doses, and the extent of dilation was 20–30% of that observed in normal arterioles [62]. In an atherosclerotic cynomolgus monkey model, coronary vasoconstrictor responses to serotonin were potentiated by atherosclerosis [63].

Chronic cocaine administration with concomitant high-cholesterol feeding attenuated endothelium-dependent relaxation responses to serotonin, suggesting cocaine can exert a direct vasoconstrictor effect on the porcine coronary microcirculation via a muscarinic mechanism in the setting of hypercholesterolemia [64]. In a primate model, peak constrictions to serotonin were markedly enhanced in a long-term hypercholesterolemic group compared with control and short-term hypercholesterolemic responses [65].

Pharmacologic targeting of these aberrant responses has received renewed attention. Matsumoto and colleagues recently reviewed three decades of evidence on altered vascular 5-HT responsiveness in diabetes models and patients, arguing that 5-HT2A blockade by sarpogrelate is mechanistically rational for diabetic macro- and microvascular dysfunction [29]. Tappia and colleagues demonstrated in streptozotocin-diabetic rats that six weeks of sarpogrelate treatment (5 mg/kg/day) attenuated hemodynamic dysfunction and restored mitochondrial oxidative phosphorylation, Ca^2+^ uptake, sarcolemmal Na^+^-K^+^-ATPase and Na^+^-Ca^2+^ exchange—effects comparable to those of insulin treatment [49]. At the population level, the SHIELD cohort (*n* = 10,778 high-risk diabetic Korean patients) found that sarpogrelate compared with aspirin for primary prevention was associated with lower net adverse clinical events (hazard ratio 0.71, 95% CI 0.57–0.88), driven principally by a 62% reduction in overall bleeding (HR 0.38, 95% CI 0.17–0.81) [48]. Overall, the mechanistic rationale, success in animal models, and established safety ratios shown by 5-HT2A antagonism is persuasive, yet insufficient at the moment. However, these data provide the strongest indication for reappraising 5-HT2A antagonism for use in diabetic vasculopathy.

### 4.2. Diabetic Alteration of Vascular Serotonin Response in Humans

A large body of evidence indicates that diabetes alters vascular responses to serotonin. Diabetic patients show significantly reduced vasodilatory capacity in response to exercise [66]. Even when subjected to methacholine challenge, patients with diabetes show persistently diminished vasodilatory capacity [67]. While multiple factors contribute, diabetic patients have increased plasma serotonin and decreased intraplatelet serotonin [68]. Animal models support these observations. Otsuka Long-Evans Tokushima fatty (OLETF) rats, a model of late-onset diabetes, show increased serotonin-mediated vasoconstriction and serotonin-mediated inhibition of voltage-gated potassium channels in vascular smooth muscle—a key contributor to normal vessel relaxation [69]. The literature also suggests increased responses through 5-HT2A and 5-HT2B receptors, although there is conflicting evidence as to whether the change reflects increased receptor expression or increased agonist sensitivity at unchanged receptor density [69,70]. Streptozotocin-induced diabetic rat models, a classic model of type 1 diabetes, show that abnormal vascular resistance may reflect broad vasomotor dysfunction across multiple vasogenic agents, with these models actually showing reduced vasoconstriction to serotonin [71]. A contemporary human cohort study of 84 pre-CABG patients measured brachial flow-mediated dilation alongside serum 5-HT, superoxide dismutase-1 and lectin-like oxidized LDL receptor-1, finding that type 2 diabetes was associated with more severe endothelial dysfunction across all measured markers, anchoring the animal data in a current human dataset [72].

## 5. Serotonin and Hypertension: Animal and Human Studies

There is considerable debate about the role of serotonin in essential hypertension. Mesenteric artery segment studies indicate similar vasogenic response curves to 5-HT between normotensive and hypertensive patients regardless of treatment status [73]. However, other work demonstrates that serotonin sensitivity in peripheral vessels is increased in elderly and hypertensive patients, specifically due to increased platelet turnover and subsequent endothelial dysfunction [74,75]. In animal models of chronic hypertension, increased platelet turnover produces a state of reduced serotonin clearance from plasma and resultant overactivation of serotonin-mediated vasogenic pathways [76]. The 5-HT2A antagonist ketanserin has been studied as a potential antihypertensive: at least one randomized study showed ketanserin increased forearm blood flow and brachial artery compliance while reducing forearm vascular resistance significantly more than metoprolol [77]. At minimum, preliminary evidence supports a role for serotonin in hypertensive states when platelet involvement is suspected and supports serotonin antagonism as a viable intervention in established hypertension.

## 6. Alterations in Vascular Responses to Serotonin Following Chronic Myocardial Ischemia

In healthy pigs, serotonin induces relaxation of endocardial and epicardial microvessels, the former slightly more so than the latter. Endothelium-denuded control myocardial microvessels demonstrate impaired responses to serotonin, indicating endothelial dependence [78]. In ischemia–reperfusion injury with impaired endothelial function, vasodilator responses to 5-HT were significantly attenuated in the porcine coronary artery occlusion model [79,80]. Relaxation responses of control myocardial arterioles to serotonin were slightly greater in subendocardial than subepicardial vessels, and subendocardial arterioles were more affected by cardioplegia than subepicardial vessels [81]. Receptor-mediated endothelium-dependent relaxation to serotonin was reduced following chronic ischemia, while chronic treatment with FGF normalized responses to serotonin (along with ADP and acetylcholine) in the collateral-dependent left circumflex region but not in the normally perfused left anterior descending region [82]. This recovery likely reflects FGF-2-mediated coronary collateral angiogenesis with restoration of endothelial nitric oxide synthase function in the collateralized region. Intracoronary serotonin increases coronary blood flow with rapid tachyphylaxis [83].

Vessels treated with a non-selective COX inhibitor (naproxen) and a selective COX-2 inhibitor (celecoxib) demonstrated increased microvascular contractile responses to serotonin [84]. In a porcine model of chronic hypertension utilizing chronic coronary constriction, microvessels in the ischemic territory supplied by the constricted vessels displayed abnormal contraction responses to serotonin, while adjacent normal coronary microvessels demonstrated smaller contractile responses to endothelin-1 and serotonin [85,86]. Precontracted arterioles dilated minimally to serotonin, which produced greater contraction in non-precontracted arterioles than in non-precontracted venules [87]. Relaxation of venules by serotonin was inhibited to varying degrees by indomethacin, methylene blue and NG-methyl-L-arginine (L-NMMA). Both methylene blue and L-NMMA produced minimal but significant contraction of non-precontracted venules, indicating modest basal release of endothelium-derived relaxing factor. These results demonstrate that coronary arterioles and venules respond differently to serotonin, with serotonin-induced vasodilation mediated in part by endothelium-derived relaxing factor and prostaglandins.

Evidence in humans on serotonergic responses to ischemia outside the context of CP/CPB is limited, reflecting the ethical challenges of subjecting human subjects to such events, but serotonin’s role in proxy events for ischemia (myocardial infarction and stroke) is well supported. Plasma sampled from the coronary arteries of patients post myocardial infarction showed increased serotonin concentrations compared with plasma from femoral blood from the same patients [88]. Serotonergic platelet responses may also contribute to ischemic event risk through platelet aggregation: individuals with the 5-HT2A T102 (T/T) genotype show higher baseline platelet aggregation than individuals without the mutation [89].

## 7. Baseline Alterations in Microvascular Tone Following Cardioplegic Ischemia/Reperfusion and Cardiopulmonary Bypass

CP/CPB significantly affects microvascular function in patients undergoing cardiac surgery. Prior work has shown a marked reduction in microvascular tone following cardioplegia in both human and animal skeletal muscle tissue, contributing to postoperative organ injury due to low perfusion [90]. Diminished post-CP/CPB microvascular tone is more pronounced in diabetic patients following cardioplegia; patients with uncontrolled hypertension show less diminished postoperative microvascular tone compared with normotensive patients [91,92]. Responses to several endogenous vasoconstrictor agents—including neuropeptide Y, vasopressin and serotonin—are markedly altered (either diminished or augmented depending on tissue bed) post-CP/CPB in patients and in animal models, with most alterations occurring in atrial rather than peripheral tissues [93]. These alterations are endothelium-dependent vascular reactivities, specifically mediated by cell surface receptor expression, weakened barrier activity, and a loss of balance between vasoconstrictive and vasodilatory mediators. The resulting vasomotor dysfunction has been shown in both animal and human models to be more pronounced in diabetes [91]. Hypertensive patients show disproportionately strong contractile responses to vasomotor agonists post-CP/CPB [91]. The 2024 EACTS/EACTAIC/EBCP guidelines synthesize current best CPB practice [94], and a recent meta-analysis of 100 vasoplegia studies reported a pooled vasoplegia incidence of 19.9% (95% CI 16.1–24.4) across cardiac surgery cohorts, quantifying the clinical scope of the problem [95].

### 7.1. Serotonin Responses in the Cardiac and Pulmonary Microcirculations Post-CP/CPB in Animal Models

Endocardial microvessel responses to serotonin were slightly more impaired by cardioplegia and ischemia–reperfusion than corresponding responses in epicardial vessels in a porcine CP/CPB model [87,96]. Notably, hypothermia during CPB caused a partial but significant reduction in the degree of impairment of relaxation responses to serotonin by inhibiting endothelium-dependent mechanisms [97]. The responses of coronary venules to serotonin remained unchanged following CP/CPB [98]. Serotonin stimulated vasodilation in control pulmonary microvessels; in the presence of the nitric oxide synthase inhibitor NG-methyl-L-arginine, the vasodilatory response was converted to a contractile response [99]. After total CPB and pulmonary reperfusion, pulmonary microvessels exhibited more significant contractile responses to serotonin compared with control vessels [99]. Indomethacin blocked the enhanced contractile responses to serotonin, suggesting a role for enhanced release of a constrictor prostanoid [100].

### 7.2. Coronary Microvascular Responses to Serotonin in Humans Following CP/CPB

In line with animal studies, previous work from our group showed that serotonin causes slight vasodilation prior to CP/CPB in human coronary microvessels that is almost completely reversed after CP/CPB in cardiac tissue [78]. Mild baseline vasodilation induced by serotonin gives way to a potent contractile response after CP/CPB that is inhibited by the selective inducible COX-2 inhibitor NS398 [101]. Expression and protein levels of COX-2 are significantly increased after CP/CPB in both human cardiac vessels and porcine pulmonary vessels [101,102]. COX-2 produces prostaglandins, which are thought to drive the serotonergic vasoconstrictor responses observed post-CP/CPB [103].

**Figure 1:**
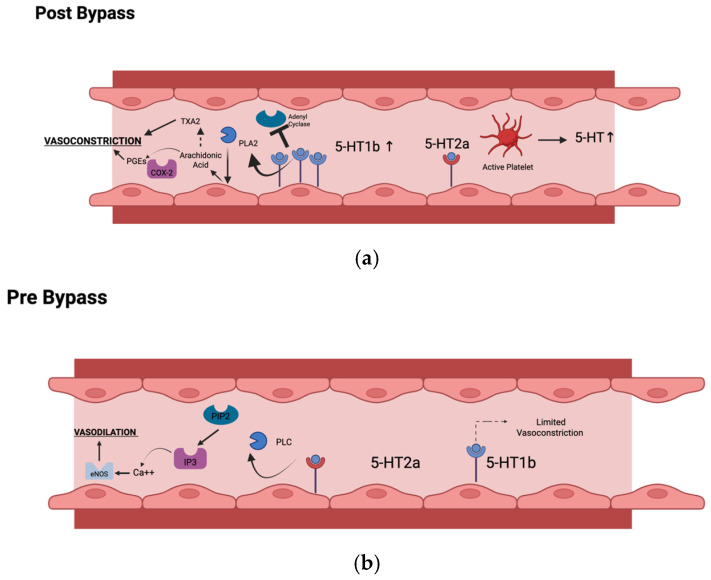
(**a**) Changes in serotonergic signaling in blood vessels occurring after cardiopulmonary bypass with cardioplegia. Prior to bypass, 5-HT2A signaling leads to a PLC-mediated cascade ending in eNOS activation and vasodilation. (**b**) Although this pathway remains activated post-CPB, the additional upregulation of 5-HT1B receptors after bypass shifts the dominant signal to a PLA2 cascade with increased prostaglandin secretion and vasoconstriction together with increased adenylyl cyclase inhibition. All of this is in the context of increased platelet-derived serotonin release, leading to a net upregulation of vasoconstriction over vasodilation.

In coronary microvessels, one proposed model for serotonergic vasomodulation centers around two main serotonin receptor subtypes: 5-HT2A elicits vasodilation, while 5-HT1B drives vasoconstriction [11,104,105]. The differential response in vasogenic tone pre- and post-CP/CPB in this model is thought to reflect this receptor balance [101,106]. This is reflected both in 5-HT2A versus 5-HT1B endothelial receptor proportions and in mean RNA and protein expression levels [26]. Addition of a 5-HT1B receptor antagonist and phospholipase A2 inhibitor—phospholipase A2 being a downstream effector of 5-HT1B—significantly decreased microvascular contraction post-CP/CPB (Figure 1) [26]. However, future studies are needed to directly assess 5-HT2A vs. 1B receptor density and expression pre- and post-CP/CPB to provide ultimate evidence for this hypothesis.

### 7.3. Role of Preexisting Diabetes in Serotonin-Mediated Vasogenic Responses Following CP/CPB

A high glucose level during CPB is an independent predictor of mortality regardless of actual diabetes status but disproportionately so in diabetic patients (odds ratio 1.20 vs. 1.1) [107]. Poorly controlled diabetic patients display more significant impairments in microvascular relaxation after cardiac surgery than non-diabetic or well-controlled diabetic patients [90,92]. Contractile responses of peripheral arterioles to 5-HT were significantly impaired following CP/CPB in both diabetic and non-diabetic patients compared with pre-CPB values, with a significantly more pronounced effect in diabetic patients [24]. There was no recorded difference prior to surgery, suggesting that the serotonergic response is specifically impaired by cardioplegia in a way that disproportionately affects diabetic patients [24]. In either case, postoperative contractile responses were blunted by 5-HT1B inhibition, corroborating prior findings implicating this receptor in post-CPB vasospasm [24]. Although these studies examined the role of distribution of 5-HT1B, previously known to be expressed in both endothelium and smooth muscle, it is worth considering that diabetes causes significant endothelial cell damage as well as downregulation of endothelial factors that promote vascular tone and motility [24,90]. The Kanuparthy 2025 review provides a comprehensive synthesis of diabetes- and hypertension-related microvascular dysfunction across multiple vasoactive agents post-CP/CPB [91].

### 7.4. Role of Preexisting Hypertension in Vasogenic Responses Following CP/CPB

Patients with poorly controlled hypertension show increased myogenic tone in coronary arterioles following cardiac surgery and increased protein kinase C alpha phosphorylation [108]. Studies in coronary vessels showed that 5-HT1B receptor expression is significantly higher in patients with poorly controlled hypertension compared with normotensive or well-controlled hypertensive patients following CP/CPB [27]. Given that this receptor had previously been implicated in microvascular contraction, follow-up studies showed that uncontrolled hypertensive patients had significantly higher microvascular contraction in response to serotonin in a dose-dependent manner [27]. These results suggest that the contribution of uncontrolled hypertension to increased coronary myogenic tone following CP/CPB may be partially mediated by 5-HT1B signaling; however it is worth noting that, as with diabetes patients, these studies are largely in limited cohorts, where studies can only be performed on microvasculature derived from consented patients. Due to the inherent limits on such studies, the majority of this mechanistic evidence, as with the original 5-HT1B hypothesis, warrants further evaluation in larger volume cohort studies and later clinical trials leveraging 5-HT1B regulatory changes if sufficient basic science evidence thresholds can be met.

## 8. Systemic Effects of Serotonin in Cardiopulmonary Bypass and Cardiac Surgery

Compared to evidence regarding vasodilatory mechanisms contributing to post-cardioplegeia cardiovascular dysfunction, which is largely endothelium-mediated, investigation into platelet-derived systemic effects of serotonin possesses sparse mechanistic evidence. Results are largely correlative in patients and warrant further exploration.

Plasma serotonin concentration is slightly increased in the systemic circulation post-CP/CPB; this has served as a justification for ketanserin prophylaxis for both peripheral and pulmonary hypertension post-CPB [109,110]. Increased serotonin levels in the peripheral and pulmonary circulations have correlated with postoperative left ventricular ejection fraction, cardiac output, systemic vascular resistance and postoperative hypertension [111]. One study in newborn lambs suggested potential cerebral endothelial dysfunction in the setting of cardioplegia, specifically under hypothermic conditions [112]. There has been at least one documented case of serotonin syndrome during CPB when other serotonergic compounds were introduced (such as methylene blue, which acts through MAO-A inhibition); while this was attributed to the presence of SSRI treatment in the patient, the previously discussed studies warrant further investigation into whether postoperative serotonin plasma concentrations may play a role in systemic disease risk [113].

However, the relationship between systemic serotonin levels and poor CPB outcomes is not a simple causal one. This can be observed through clinical use of ketanserin as mentioned above, which acts as a 5-HT2A receptor antagonist [114]. Early studies of ketanserin prophylaxis demonstrated significant effects on lowering blood pressure and reducing serotonin-mediated platelet aggregation, making it useful for controlling vasospastic disorders and platelet activity [115]. Thrombocytopenia was still observed in these patients, suggesting that its mechanism may be driven by other factors in CPB (mechanical destruction) and is not dependent on serotonin. The antihypertensive effects of ketanserin may also derive from its α1-adrenergic receptor antagonism, suggesting that the primary role of serotonin in CPB-related cardiac events may be limited to platelet activation [116].

In contrast to direct receptor antagonism, the usage of Iloprost, an analog of prostacyclin PGI2, may offer further evidence that serotonin’s perioperative effects on hemodynamics and thrombosis are more likely to affect short-term primary outcomes such as arterial blood pressure than they are to improve long-term post-surgical outcomes. The major therapeutic use for Iloprost has been its strong selective vasodilatory effects on the pulmonary circulation which spares the systemic circuit when inhaled [117]. Although not a direct antagonist of serotonin, Iloprost acts indirectly on serotonin activity by sequestering it in platelets to reduce plasma levels of serotonin [118]. However, while observations of Iloprost use in the setting of cardiac surgery suggests that Iloprost may reduce the risk of heparin-induced thrombocytopenia (HIT), other secondary outcomes such as time-to-extubation were unaffected [119,120,121,122]. In addition, usage of intravenous Iloprost in the ICU for patients with septic shock did not correspond to reductions in mean daily SOFA scores or mortality [123,124]. If Iloprost does effectively sequester serotonin within platelets and opposes its vasoconstrictive effects, then its failure to affect outcomes would be in line with previous observations that plasma serotonin levels are not correlated with postoperative hypertension following revascularization [125]. Iloprost instead seems to show a greater efficacy when used outside of the operating room in the management of chronic pulmonary hypertension [118]. At the very least, Iloprost use did not demonstrate an increased risk of adverse events, so the potential application of its serotonergic activity remains as a viable field of study [120].

Additional insight comes from patients chronically taking SSRIs before cardiac surgery. SSRIs deplete intraplatelet serotonin by blocking platelet serotonin uptake, creating a long-term state of altered serotonin handling and altered in vitro platelet responses to typical platelet activators [126]. Single-variate analysis of SSRI use and CPB outcomes appeared to demonstrate an increased risk of adverse events such as blood transfusions and inotrope use [127]. However, multivariate analysis controlling for confounders demonstrated that SSRI use did not increase rates of major bleeding, reoperation for hemorrhage, mortality or other major adverse events despite theoretical concerns that reduced intraplatelet serotonin might impair hemostasis [127]. Certain postoperative outcomes such as prolonged mechanical ventilation and ICU stay were observed but may be explained by underlying conditions [128]. Although there is no strong evidence that SSRIs increase the risk of adverse outcomes in CPB alone, SSRI use may be a conditional modifier given a patient’s psychiatric and genetic profile. Philips-Bute and colleagues found that while SSRI use alone did not predict increases in postoperative cardiac events, depression and the presence of a dominant allele in the serotonin transporter gene both increased the risk of a cardiac event up to 12 months post-operation [129]. Collectively, these SSRI outcome studies function as a natural experiment demonstrating that baseline serotonergic modulation does not amplify CPB-related systemic complications, suggesting that acute serotonin surges during CPB—not chronic serotonin levels—are the dominant driver of postoperative vasomotor dysfunction and hemodynamic disturbance.

Beyond vasomotor dysfunction, the effects of serotonin on primary hemostasis in CPB are observed through its involvement in heparin-induced thrombocytopenia (HIT). Heparin exposure has been linked to the presence of heparin-dependent antibodies, and the large quantity of heparin used in CPB is correlated with increased postoperative detection of those antibodies [130]. However, this dramatic increase in antibodies does not have a proportional rise in thrombocytopenia or thrombosis, possibly because of variations in antibody pathogenicity [130]. Rather than ELISA tests, serotonin release assays (SRA)—which detect a subject’s plasma’s ability to induce serotonin release in foreign donor platelets—are typically used as the functional gold standard for HIT confirmation [131]. In CPB, SRA alone is a low-specificity test because while many patients test positive (defined as >20% release) and many develop thrombocytopenia, CPB-related consumption of platelets may be the main contributor to thrombocytopenia, and many of those patients do not clinically develop thrombosis [132]. Given the low incidence of HIT even in a post-CPB setting, it may be more useful to interpret a positive SRA test in the context of other tests to understand the individual patient’s clinical risk. In the context of a D-dimer test, SRA may still indicate the presence of pathogenic prothrombotic antibodies, though for asymptomatic patients this may only represent subclinical thrombosis [132]. Close monitoring of platelet count may also inform interpretation of a positive SRA, as a biphasic platelet count pattern—normal recovery followed by a secondary drop after postoperative day 5—was highly predictive of a positive SRA, suggesting an identifiable delayed phase of serotonin-induced platelet activation [133]. For patients with previous HIT undergoing cardiac surgery, therapeutic plasma exchange (TPE) has been used prophylactically, and anecdotal evidence suggests limited TPE is enough to lower antibody concentrations enough to significantly lower serotonin release in response to heparin [134]. Heparin concentrations used during CPB (1–3 IU/mL) after TPE suppressed the formation of PF4–heparin–IgG complexes and reduced serotonin release, suggesting a mechanism by which intraoperative heparin can transiently reduce platelet-derived serotonin release in pre-treated patients [134].

## 9. Discussion of Limitations

This review is a selective narrative synthesis rather than a systematic review, and therefore may reflect selection bias based on the available literature and author judgment. Many mechanistic insights derive from porcine and rodent models, which differ from human vascular physiology, receptor-expression patterns, and microvascular architecture, limiting direct translational interpretation. As a result, mechanistic models proposed here—particularly those involving receptor-subtype dominance, prostanoid coupling, and tissue-bed-specific vasomotor patterns—should be interpreted as biologically plausible frameworks supported by preclinical evidence rather than definitive human mechanisms. Direct human validation remains limited by ethical constraints, heterogeneity in surgical tissue sampling, and the lack of receptor-selective pharmacologic tools. The studies summarized here employ heterogeneous vascular reactivity assays—including wire myography, pressure myography, isolated microvessel preparations, and flow-mediated dilation—which are not directly comparable and may contribute to variability in reported serotonergic responses. Interpretation of receptor-specific mechanisms is further constrained by limited pharmacologic specificity, particularly the difficulty of distinguishing 5-HT1B from 5-HT1D signaling with currently available antagonists. Finally, while we discuss potential therapeutic implications, these are grounded primarily in mechanistic plausibility, preclinical models, and observational clinical data, with relatively few randomized interventional trials available to confirm causality.

## 10. Summary and Future Directions

Serotonin is a vasoactive tryptophan-derived monoamine that plays a significant role in regulating vasomotor tone. Fifteen serotonin receptors mediate slightly different downstream signaling effects in different vascular beds. Abnormal serotonin activity may exacerbate microvascular dysfunction in a variety of cardiometabolic disease states including diabetes, hypertension, chronic myocardial ischemia and the post-CP/CPB state. Abnormal serotonin responses may reflect increased plasma serotonin levels, changes in receptor expression, or changes in downstream signaling. Preexisting diabetes and hypertension predispose patients to more significant vascular dysfunction following CP/CPB, creating an additive effect on microvascular dysfunction.

Future studies will need to clarify several gaps. First, more definitive links between 5-HT receptor genotypes and cardiometabolic disease states are needed. Second, direct quantification of changes in serotonin receptor density in the context of chronic myocardial ischemia, diabetes, hypertension and CP/CPB is required to confirm the receptor-expression hypothesis. Third, the mechanistic relationship between prostacyclin pathway modulation and serotonergic signaling deserves further dissection. Fourth, large clinical studies testing the impact of serotonin-signaling blockade on protecting microvascular function in diabetes, hypertension and ischemic injury are needed. One natural starting point would be clinical trials testing 5-HT1B antagonists in the postoperative management of patients with poorly controlled hypertension undergoing cardiac surgery to assess whether the receptor-level findings translate to improved morbidity and mortality. The recent body of evidence on 5-HT1B in post-CPB vasospasm—from Robich and colleagues [26], Sabe and colleagues [108], and Harris and colleagues [27]—makes this receptor the highest-priority perioperative target. To this end, 5-HT1B antagonists remain to be developed that can safely enter human trials. Discontinued candidates such as AZD3783 and Elzasonan have previously seen attempts at use in human trials, whereas classically described antagonists (SB-224289, SB-216641, etc.) in a preclinical setting may yet prove to inform development of in vivo compatible analogs in humans [135,136,137,138].

Independently, peripheral 5-HT modulation (Tph1 inhibition; sarpogrelate-style 5-HT2A antagonism; peripherally restricted 5-HT2B antagonism) merits continued translational development for chronic cardiometabolic disease. One possible study design would be a randomized, double-blind, placebo-controlled trial of perioperative sarpogrelate in poorly controlled hypertensive patients undergoing isolated CABG with CP/CPB. Primary endpoints could include the incidence and severity of post-CPB vasospasm or vasoplegia, quantified through vasopressor requirements, hemodynamic stability, and lactate clearance. Secondary endpoints may include microvascular reactivity, postoperative myocardial performance, ICU length of stay, and safety outcomes such as bleeding or arrhythmias. Such a study would provide the first interventional test of serotonergic pathway modulation in a high-risk surgical population and directly address the mechanistic hypotheses synthesized in this review. Ultimately, finding safe and effective ways of modulating serotonergic signaling in cardiometabolic disease offers a path forward for improving outcomes in patients undergoing cardiac surgery and patients with chronic cardiometabolic disease alike.

## Figures and Tables

**Table 1:** Cardiovascular-relevant serotonin receptors and their primary vascular actions.

Receptor	G-Protein Coupling	Primary Vascular Effect	Tissue Bed	Key References
5-HT1A	Gi/o	Vasodilation	Coronary, peripheral	[22,23]
5-HT1B	Gi/o	Vasoconstriction	Coronary, peripheral; upregulated post-CPB	[24,25,26,27]
5-HT1D	Gi/o	Vasoconstriction	Peripheral	[28]
5-HT2A	Gq/11	Coronary vasodilation; peripheral vasoconstriction	Coronary, peripheral, renal	[29,30]
5-HT2B	Gq/11	Vasoconstriction; smooth-muscle mitogenesis; fibrogenesis	Pulmonary, peripheral, hepatic	[31,32]
5-HT4	Gs	Positive inotropy and chronotropy	Atrial myocardium	[20]
5-HT7	Gs	Vasodilation (predominantly venous)	Venous, peripheral	[21]

## Data Availability

No new data were created or analyzed in this study. Data sharing is not applicable to this article.
